# Performance Analysis of a CSFH-Based Microgripper: Analytical Modeling and Simulation

**DOI:** 10.3390/mi13091391

**Published:** 2022-08-25

**Authors:** Teferi Sitotaw Yallew, Nicola Pio Belfiore, Alvise Bagolini, Maria F. Pantano

**Affiliations:** 1 Department of Civil, Environmental and Mechanical Engineering, University of Trento, 38123 Trento, Italy; 2 Micro Nano Facility, Fondazione Bruno Kessler, 38123 Trento, Italy; 3 Department of Engineering, University of Roma Tre, 00146 Rome, Italy

**Keywords:** micro-manipulation, MEMS, FEM, cell characterization

## Abstract

Microgrippers are promising tools for micro-manipulation and characterization of cells. In this paper, a biocompatible electro-thermally actuated microgripper with rotary capacitive position sensor is presented. To overcome the limited displacement possibilities usually provided by electrothermal actuators and to achieve the desired tweezers output displacement, conjugate surface flexure hinges (CSFH) are adopted. The microgripper herein reported can in principle manipulate biological samples in the size range between 15 and 120 µm. A kinematics modeling approach based on the pseudo-rigid-body-method (PRBM) is applied to describe the microgripper’s working mechanism, and analytical modeling, based on finite elements method (FEM), is used to optimize the electrothermal actuator design and the heat dissipation mechanism. Finally, FEM-based simulations are carried out to verify the microgripper, the electrothermal actuator and heat dissipation mechanism performance, and to assess the validity of the analytical modeling.

## 1. Introduction

Mechanical manipulation and characterization of cells are fundamental activities in biological and biomedical research. Due to the microscale size and highly fragile nature of the involved materials, conventional cell manipulation and characterization techniques do not provide sufficient accuracy and performances [1].

Microelectromechanical systems (MEMS) technologies are helpful in the development of new and efficient tools for cell manipulation and characterization due to their capacity to downscale size and forces. Different MEMS platforms have been built with different geometries [2] and different actuating and sensing strategies [3] to manipulate micro-objects and to measure cellular forces or cell deformation [4,5,6], to name but a few. Specifically, microgrippers are MEMS devices used to pick, transport, and place a cell at the desired testing location. Additionally, they can integrate force sensors to measure and control the grasping force, preventing cell damage and enabling characterization of the mechanical properties of cells [7].

During design, there are some specific gripper’s characteristics that have to be considered for the proper operation of the device. Those include actuator type, power consumption, geometry of the compliant mechanism and of gripping jaws, displacement and force range available at jaws, and material type [8].

In regards to the actuation system, there are different types of micro-actuators that have been proposed. The most commonly used are electrostatic, electrothermal, electromagnetic, and piezoelectric actuators [9]. Electrothermal actuators are often preferable when compactness, low voltage, large output force, and stability are of primary importance. Typically, an electrothermal actuator consists of a number of v-shaped beams connected to the substrate at anchor regions. The voltage applied across the anchors produces a current flow through the beams that, in turn, heats the beams due to the Joule effect. The increase in temperature causes the thermal expansion of the beams, which move a central shuttle. The relatively high operation temperature characterizing thermal actuators can make them robust against moisture formation, for example, when manipulation and characterization of biological materials are required in humid environment. However, the induced high temperature during operation may restrict their application in manipulating temperature sensitive materials. This problem can be handled by adding heat sink beams [10,11,12] to dissipate the heat to the surrounding fluid or the device frame. In the case of a MEMS-based gripping platform, heat sink beams can be placed in cascade with the actuator to provide temperature reduction towards the grippers, as proposed by the authors in a previous study [13].

Although electrothermal actuation can typically provide relatively small displacements [14,15], a magnification stage can be added between the actuator and the gripper. In general, amplification mechanisms based on complaint structures are gaining importance in MEMS applications, especially in all those situations where motion precision, reliability, accuracy, and compactness are needed. For displacement amplification designs, micro-flexures and hinges present many advantages, such as motion repeatability, and absence of backlash and lubrication [16].

Compliant mechanisms have the ability to modify their configuration as a consequence of an elastic deformation of their flexure hinges. The latter can be classified as primitive flexures (long flexible beams, notch type hinges) and complex flexures (combination of two or more primitive flexures) [17]. Recently, the conjugate surface flexure hinge (CSFH) [18] has been proposed, which can work as a complex or a primitive flexure, depending on the load conditions. This flexure hinge allows the overcoming of typical limitations of the above-mentioned primitive and complex flexures, such as reduced capability in terms of motion/force transmission, and the center of the relative rotation between two adjacent links not being fixed during the relative motion of the rigid links [19].

Furthermore, to make the gripper operation safe and to prevent damage to the grasped objects, it is highly recommended to use sensing mechanisms. Depending on the final application and the sample to be manipulated, it is desirable to equip a microgripper with different types of sensors. Those can be piezoresistive, piezoelectric, capacitive, electrothermal, and vision-based. From the listed sensors, capacitive readout is the standard for inertial MEMS feedback owing to low energy consumption, higher sensitivity, good frequency response, high spatial resolution, large dynamic range, and imperceptibility to environmental changes [20].

Many researchers have developed different kinds of microgrippers to achieve large displacements and high amplification ratios in addition to increasing their degree of freedom [21,22,23,24,25]. Moreover, it is observed that most of the high amplification ratio microgrippers have relatively low tweezer displacement and are limited to operate with one degree of freedom (DOF) [25].

Therefore, the objective of the present work is to design a novel MEMS microgripper with integrated actuation and position sensing capabilities. Pseudo-rigid-body (PRB) modeling approach is used to design the microgripper, and analytical modeling, based on the finite elements method (FEM), is employed to optimize the electrothermal actuator and heat dissipation mechanism. Moreover, 3D structural and thermal simulations are performed to verify the validity of the theoretical modeling approaches. Our microgripper adopted fabrication oriented design specifically for standard MEMS technology. In particular, silicon-on-insulator (SOI) wafer with deep reactive ion etching (DRIE) technique has been considered. Due to its designed application and foreseen experimental application, the initial requirements and restrictions to the design are: (i) Need for humid environment compatibility, as required for in vitro manipulation of biological samples, which is addressed by the implementation of thermomechanical actuation rather than the electrostatic one [26,27]; (ii) target displacement range as large as 52.5 µm for each gripper arm (i.e., to provide more than 100 µm gripper offset since cells vary in size; therefore, a microgripper with wide gripping range is desired); (iii) target tweezer maximum acceptable temperature of 22 °C [28]; (iv) minimum feature size of 6 µm; and (v) minimum gap size of 2 µm due to MEMS fabrication technology constraints on a 25 µm thick SOI device layer.

## 2. Overall Design of the Microgripper

**Figure 1:**
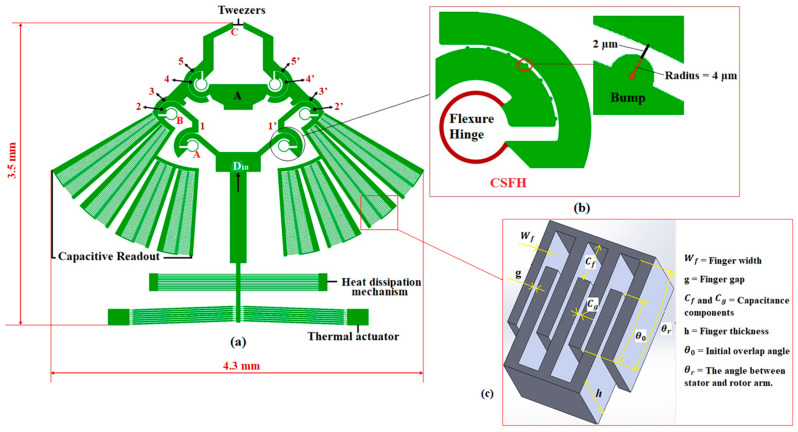
(**a**) Microgripper model with all its main components; (**b**) layout of a conjugate surface flexure hinge (CSFH) with a series of bump-shaped structures; (**c**) capacitive readout.

The model of our proposed microgripper is presented in this section. Figure 1 shows all the components of our microgripper, which consists of a pair of gripping tweezers driven by an electrothermal actuator, a heat dissipation mechanism, and an integrated rotary capacitive position sensor (Supplementary Video S1). To convert the displacement delivered by the thermal actuator into a gripping mechanism, conjugate surface flexure hinges (CSFH) are used herein.

In a CSFH mechanism, a series of bump-shaped structures are usually added (Figure 1b). These structures act as a mechanical constraint and are used to limit the movement of the hinge’s rotation center and maintain the interdigitated capacitive readout fingers in place [29].

### 2.1. Working Principle of the Microgripper

The CSFH includes a thin curved beam, as a flexible element, and a pair of conjugate surfaces (2 and 3, 4 and 5, 2′ and 3′, 4′ and 5′). These flexure hinges enable displacement to be transmitted and amplified. In our device, six CSFHs are introduced to achieve the desired opening of the tweezers.

As shown in Figure 1a, the actuation force, which is delivered by the thermal actuator, develops an upward force on the links (1 and 1′) and, as a consequence, the conjugate surfaces (2 and 2′) rotate in the clockwise direction with the flexure hinge around the center of rotation of the revolute conjugate surfaces and the flexible hinges. Simultaneously, the other end of the flexure hinges produces a reaction force to resist the rotation. This reaction force pushes the bottom edges of the links (3 and 3′) up and then the conjugate surfaces (5 and 5′) rotate in the counter clockwise direction around the center of rotation of the revolute conjugate surfaces and the flexure hinges. Eventually, this rotation develops a gripping force at the tip of the tweezers.

### 2.2. Modeling of the Microgripper

#### 2.2.1. Kinematic Modeling

**Figure 2:**
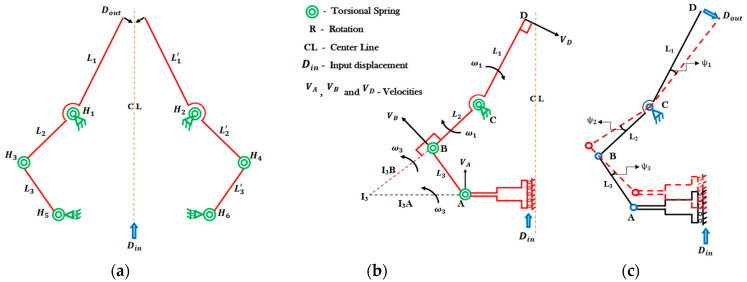
(**a**) Pseudo-rigid-body equivalent model of the microgripper; (**b**) velocity vector diagram of the microgripper; (**c**) angular changes of the microgripper.

Based on a pseudo-rigid-body-method equivalent mechanism [30], the flexure hinges $H_{1}$–$H_{6}$ can be seen as equivalent to rotational springs, while the connecting links can be considered as rigid members (Figure 2a–c). The input displacement to the whole microgripper is $D_{in}$, which is provided by the actuator; $D_{out}$ is the output displacement of the grasping tweezers of the microgripper.

The kinematic analysis is performed on half of the microgripper due to its symmetrical configuration and the velocity vector diagram is shown in Figure 2b. From the velocity vector diagram, the instantaneous centers of the corresponding links can be determined.

The instantaneous velocities of points *A*, *B*, and *D* can be obtained as follows (Figure 2b):

(1) $$V_{A}=\;\omega_{3}\times I_{3}A$$

(2) $$V_{B}=\;\omega_{3}\times I_{3}B$$

(3) $$\omega_{3}=V_{A}/I_{3}A=V_{B}/I_{3}B$$

where $\omega_{3}$ is the angular velocity of link 3, $I_{3}A$ and $I_{3}B$ are the relative positions from points *A* and *B* to the instantaneous center $I_{3}$, respectively.

By considering Figure 2, the following relationship between the velocity at point *B* and *D* can be derived as:

(4) $$V_{B}=\;\omega_{1}\times L_{2}$$

(5) $$V_{D}=\;\omega_{1}\times L_{1}$$

(6) $$\omega_{1}=V_{B}/L_{2}=V_{D}/L_{1}$$

where $\omega_{1}$ is the angular velocity of links 1 and 2, $L_{1}$ and $L_{2}$ are the lengths of the corresponding links.

By considering Equations (3) and (6), we achieve:

(7) $$V_{A}/V_{B}=I_{3}A/I_{3}B$$

(8) $$V_{B}/V_{D}=L_{2}/L_{1}$$

Combining Equations (7) and (8) and calculating $V_{D}/V_{A}$, which represents $V_{out}/V_{in}$,

(9) $$V_{D}/V_{A}=\;L_{1}/L_{2}\times I_{3}B/I_{3}A$$

Therefore, the amplification ratio [31] can be computed as,

(10) $$R=\;D_{out}/D_{in}\approx \;V_{out}/V_{in}$$

Additionally, considering that during the kinematic modeling, only half of the microgripper is considered since the microgripper has a symmetrical configuration, the amplification ratio of the overall microgripper structure can be computed as:

(11) $$R_{tot}=2\;\times V_{D}/V_{A}=\;2\;\times \left(L_{1}/L_{2}\;\right)/\left(I_{3}A/I_{3}B\;\right)$$

From Expression (11), the amplification ratio is only related to the geometrical parameters of the microgripper. In the following, we investigate the static modeling of the microgripper to describe the force-deflection relationship of the flexure hinges.

#### 2.2.2. Input Stiffness of the Microgripper

An important parameter which qualifies the performance of a compliant mechanism is the input stiffness of the mechanism [32]. Therefore, input stiffness analysis is carried out to obtain the relationship between force and displacement of the microgripper.

Input stiffness is defined as the ratio of the input force at the shuttle of the microgripper ($F_{in}$) to the displacement in the axial direction ($D_{in}$). Due to the effect of the input force, there is a formation of torque at the flexure hinges, and the torque $M_{i}$ generated at the rotational center of the flexure hinges can be obtained as [33]:

(12) $$M_{i}=-K_{i}\Phi_{i}\;i=A,\;B\;and\;C$$

where $K_{i}$ is the stiffness of the i-th flexure hinges and $\Phi_{i}$ is its rotation angle. The negative sign indicates that the moment has an opposite direction to the rotational motion of the flexure hinge. Neglecting inhomogeneity and anisotropy in silicon microstructures and the corresponding stiffness matrix, and considering linear elastic beams with uniform, rectangular cross-section, and assuming that the bending moment is constant, the stiffness of the flexure hinges ($K_{i}$) can be obtained as [18]:

(13) $$K_{i}=\frac{EI}{{\theta^{\prime }}_{i}r}=\frac{Etw^{3}}{12{\theta^{\prime }}_{i}r}i=A,\;B\;and\;C$$

where E is the Young’s modulus, r is the flexure hinge radius, $\theta^{\prime }$ is the initial angle of the flexure hinges, and I is the moment of inertia of the cross-section, with I = $\frac{tw^{3}}{12}$ [34], t and w as the thickness and width, respectively.

To derive the input stiffness of the microgripper, the Castigliano’s theorem is adopted [25,30,31,35]. By considering the PRBM of the microgripper with input forces on the shuttle of the microgripper ($F_{in}$), output forces at the tip of the tweezer ($F_{out}$), and torques at each joint, the total virtual work of the system, $\delta W_{sys}$, can be written as:

(14) $$\delta W_{sys}={\vec{F}}_{in}\cdot \delta {\vec{D}}_{in}+{\vec{F}}_{out}\cdot \delta {\vec{D}}_{out}+\sum_{i=A}^{C}{\vec{M}}_{i}\cdot \delta {\vec{\Phi }}_{i}\;i=A,\;B\;and\;C$$

Based on the principle of virtual work,$\;\delta W_{sys}$ = 0 and, since ${\vec{M}}_{i}=-K_{i}\cdot \delta {\vec{\Phi }}_{i}$; therefore, Equation (14) can be obtained as:

(15) $$F_{in}D_{in}-F_{out}D_{out}-\sum_{i=A}^{C}K_{i}\Phi_{i}^{2}=0$$

Recalling Expression (11), after substitution and re-arrangement of parameters, Equation (15) can be obtained as:

(16) $$F_{in}=R_{tot}F_{out}+\frac{2U}{D_{in}}$$

where,

(17) $$U=\frac{1}{2}\sum_{i=A}^{C}K_{i}\Phi_{i}^{2}$$

During the micromanipulation of micro-objects, the grasping procedure includes closing both tweezers to approach and grasp the object, and firmly holding the object. Before the tweezers contact with the object, the output force $F_{out}$ is equal to zero, and Equation (16) can be obtained as:

(18) $$F_{in}=\frac{2U}{D_{in}}$$

where U is the deformation energy, $F_{in}$ and $D_{in}$ are the input force and the input displacement, respectively.

For a small input displacement $D_{in}$, the rotational angles $\Phi_{A}$, $\Phi_{B}$, and $\Phi_{C}$ of the flexure hinges *A*–*C* can be obtained as (Figure 2c):

(19) $$\Phi_{A}=\Psi_{3}=\frac{D_{in}}{I_{3}A}$$

(20) $$\begin{array}{c} \Phi_{B}=\Psi_{3}+\Psi_{2}=\Psi_{3}+\Psi_{3}\left(\frac{I_{3}B}{L_{2}}\right)=\Psi_{3}\left(1+\frac{I_{3}B}{L_{2}}\right)\\ =D_{in}\left(\frac{1}{I_{3}A}+\frac{I_{3}B}{I_{3}AL_{2}}\right) \end{array}$$

(21) $$\Phi_{C}=\Psi_{1}=\frac{D_{in}R}{\;L_{1}}=\Psi_{2}$$

where $\Psi_{1}$, $\Psi_{2}$, and $\Psi_{3}$ are the angular changes of the links *CD*, *BC*, and *AB*, respectively.

Substituting Equations (19)–(21) into (17) yields:

(22) $$U=\frac{1}{2}\left(\frac{K_{A}D_{in}^{2}}{I_{3}A^{2}}\right)+\frac{1}{2}\left(K_{B}{\left(\;D_{in}\left(\frac{1}{I_{3}A}+\frac{I_{3}B}{I_{3}AL_{2}}\right)\right)}^{2}\right)+\frac{1}{2}\left(K_{C}{\left(\;\frac{D_{in}R}{L_{1}}\right)}^{2}\right)$$

Substitute Equation (22) into (18), and the input force can be obtained as:

(23) $$\begin{array}{c} F_{in}=(\frac{K_{A}}{I_{3}A^{2}}+K_{B}\;\left(\frac{1}{I_{3}A^{2}}+\frac{2I_{3}B}{I_{3}A^{2}L_{2}}+\frac{I_{3}B^{2}}{I_{3}A^{2}L_{2}{}^{2}}\right)\\ +K_{C}\;\left(\;\frac{R^{2}}{L_{1}^{2}}\right)\;)\;D_{in} \end{array}$$

The input stiffness of the microgripper can be derived as:

(24) $$K_{in}=\frac{F_{in}}{D_{in}}=\left(\frac{K_{A}}{I_{3}A^{2}}+K_{B}\;\left(\frac{1}{I_{3}A^{2}}+\frac{2I_{3}B}{I_{3}A^{2}L_{2}}+\frac{I_{3}B^{2}}{I_{3}A^{2}L_{2}{}^{2}}\right)+K_{C}\;\left(\;\frac{R^{2}}{L_{1}^{2}}\right)\;\right)$$

### 2.3. Analytical Modeling of V-Shaped Thermal Actuator and Heat Sink Beams

The actuator consists of v-shaped stepped beams and heat sink beams, whose behavior will be studied in the following subsections.

Based on the literature [36], the mechanical behavior of the electrothermal actuator can be analytically derived by considering the following assumptions: (i) The average temperature increase in the inclined beams of the electrothermal actuator is known; (ii) the central shuttle is rigid and not affected by the temperature increase; (iii) small strains and displacements are considered; and (iv) the shear deformation of the beams is negligible.

The thermal actuator that is considered in the design of our microgripper consists of pairs of stepped beams connected to the substrate and a central shuttle, and it is used to drive the microgripper. Each beam has a thinner region at both ends and a thicker central part. The thinner parts are important to reduce the stiffness and enable a wider movement range, as it will be shown in the following. Despite the fact that it is widely adopted [37,38], there is no exhaustive analytical model available in the literature for this stepped beam thermomechanical actuator.

Therefore, we report for the first time a complete analytical model of this actuator. In the proposed model, the small deformation hypothesis is adopted for both lateral bending and axial deformation of the beams.

**Figure 3:**
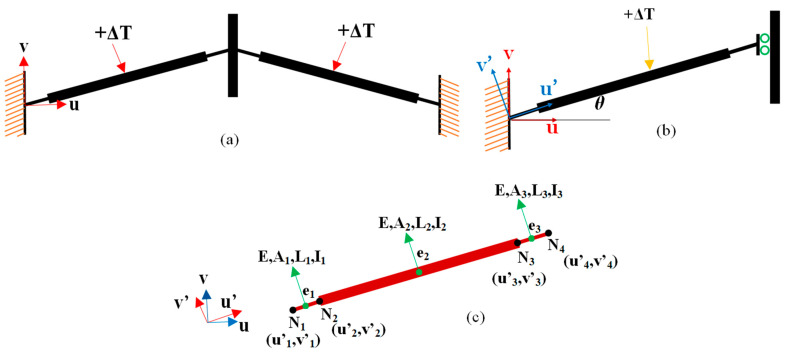
(a) Schematic of a pair of stepped inclined beams subjected to an average increase in temperature (ΔT). Each beam has a thicker and longer element in the center, and two thinner and shorter beams at the ends; (**b**) equivalent mechanical representation of a single beam in a local reference frame; (**c**) single inclined beam of the thermal actuator modeled by four nodes and three elements. Elements 1 and 3 correspond to the short beams that connect the central beam (element 2) to the anchor (left end) and shuttle (right end), respectively.

Let us consider a single inclined beam; this can be modeled by three elements ($e_{1}$,$\;e_{2}$,$\;e_{3}$), with length $L_{1}$, $L_{2}$, and $L_{3}$, respectively, and four nodes in total (Figure 3c).

The displacement at node 4 in the *v*-direction, $v^{\Delta T}$, due to an average temperature increase in ΔT along the beam, can be derived analytically (see Equation (A21) in the Appendix) according to the following procedure: (i) Discretize the inclined beam structure into its elements. Additionally, both lateral bending and axial deformation of the beams are considered. These considerations show that the beam element is treated as a frame element; (ii) compute the elastic stiffness matrix in a local reference frame ($u^{\prime },v^{\prime }$); (iii) transform the local stiffness matrix to global stiffness matrix by means of a rotation matrix; (iv) assemble the element matrices; and (v) impose the boundary conditions in the global matrix to find the displacements at each node (see Appendix A for details).

Displacement $v^{\Delta T}$ is dependent on geometrical quantities, such as the elements lengths, cross-sectional area, moment of inertia, the beam angle, the coefficient of thermal expansion of the beam material, and the Young’s modulus of the material.

The response of two inclined beams subject to an external force (F) applied to the central shuttle along the *v*-direction, can be obtained similarly. In particular, the analytical expression for the displacement at node 4, $v^{F}$, due to an external force (see Equation (A22) in the Appendix A) can be obtained starting from a similar governing system of equations (14 reported in the Appendix A), where the thermal load on the right side (i.e., αΔTEA is the thermal expansion force of the beams [39] is substituted with external force (F/2).

Then, ratio $K_{A}$ = $F/v^{F}$ represents the stiffness of one v-shaped thermal actuator beam; in which the quantity multiplied by the number, *m*, of v-shaped beams provides the overall stiffness of the thermal actuator (see Equation (A23) in the Appendix A).

In the case that the v-shaped thermal actuator beam is subjected to both a temperature increase (ΔT) and an external force (F), the displacement can be obtained as:

(25) $$v_{4}^{\Delta T+F}=v_{4}^{\Delta T}+v_{4}^{F}$$

**Figure 4:**
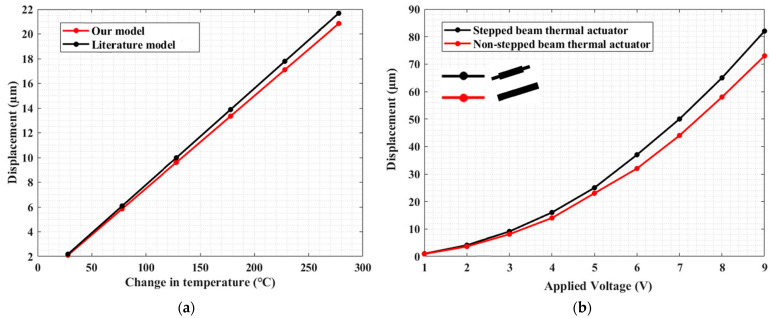
(a) Displacement vs. temperature change in one inclined thermal actuator beam with uniform cross-section modeled through a literature model [36] and our model at a tilt angle of 2°; (**b**) comparison between non-stepped beam thermal actuator and stepped beam thermal actuator in terms of displacement when the actuator is biased with 1–9 V.

To check the effectiveness of our model, we considered a thermal actuator beam with uniform cross-section. In this case (Figure 4a), we compared the results in terms of delivered displacement at varying temperature increase, obtained from our model with the ones that can be derived from the following literature model [36]:

(26) $$U^{\Delta T}=U_{y}^{A}=\alpha \Delta Tl\frac{\sin\theta }{\left(\sin^{2}\theta +\cos^{2}\theta \left(\frac{12I}{Al^{2}}\right)\right)}$$

In Figure 4a, the displacements delivered by the actuator beam obtained from both approaches have a good match with a maximum difference of ~4% at 278 °C. This result shows that our model can be effectively used for the analysis of non-stepped beam actuators, as well.

Then, we compared the performance of a stepped actuator beam with respect to a non-stepped one.

Figure 4b shows the displacement along the v-direction at the central shuttle as a function of the applied voltage for a non-stepped actuator and a stepped actuator with a ratio between the lengths of the external and the central region equal to 40/1150. The plot shows that the stepped beam thermal actuator performs better than the classical non-stepped beam thermal actuator in terms of displacement delivery, with the produced displacement to be enhanced by up to 1.12× at 9 V; this also indicates that the stepped beam thermal actuator is less stiff than the classical one.

To achieve the intended application of the microgripper, we need to meet some design goals:

1. Input displacement of ~39 µm to achieve the desired output displacement of each tweezer (52.5 µm).
2. Ambient temperature (22 °C) at the tweezer region.

From Equations (A21) and (A23) in the Appendix A, it is seen that the displacement and stiffness of the thermal actuator when unconstrained by heat sink beams depends on the beam lengths $\left(L_{1},\;L_{2},\;\mathrm{and}\;L_{3}\right)$, the beam angle (θ), change in temperature (Δ*T*), and the cross-sectional area of the beams ($A_{1},\;A_{2},\;\mathrm{and}\;A_{3}$).

**Figure 5:**
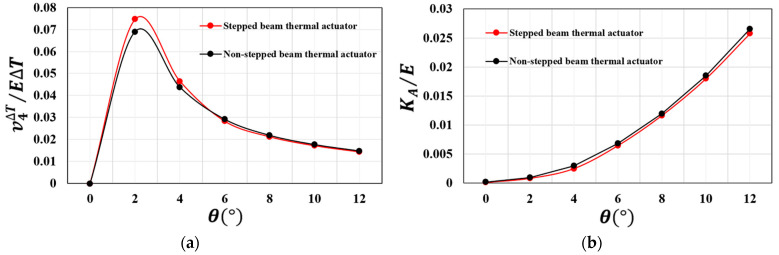
(**a**) Displacement as a function of the inclined beam angle; (**b**) stiffness as a function of the inclined beam angle.

Figure 5a shows the stepped and non-stepped beam thermal actuator displacement provided by Equation (A21) in the Appendix A as a function of the stepped and non-stepped beam inclination angle. It is seen that the displacement increases with small angles in the range of θ ≤ 2°.

Figure 5b shows the stepped and non-stepped beam thermal actuator stiffness provided by Equation (A23) in the Appendix A as a function of the stepped and non-stepped beam inclination angle. The plot shows that the actuator stiffness increases with the beam angle. In light of these results, our microgripper was designed with v-shaped beams inclined by 2°, thus with high displacement capability and reduced stiffness.

**Figure 6:**
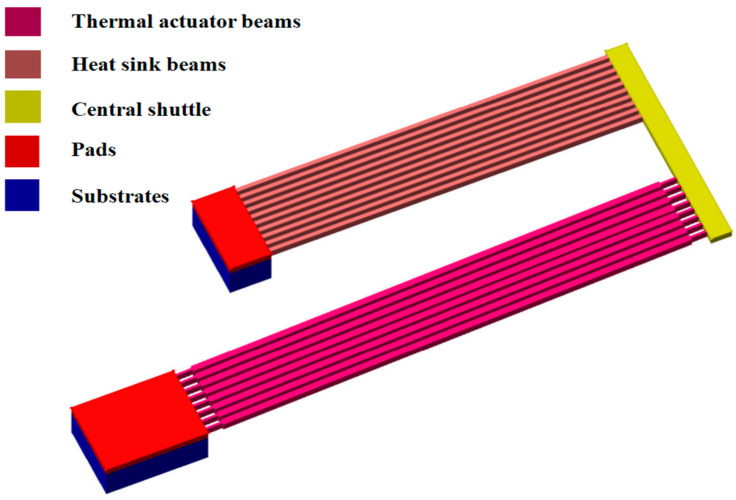
Connection of heat sink beams to the substrate and the actuator shuttle.

Then, to manage the temperature increase produced by the thermal actuator, we can consider the presence of heat sink beams located between the thermal actuator and the tweezers (Figure 1). As we see from Figure 6 (half section of heat dissipation and thermal actuator model), at the left side of the heat sink beams, there is a clamp (since the beam is anchored to the substrate), while at the right side, the shuttle acts as a slider.

Heat sink beams are subject to transverse loading that produces significant bending effects.

The displacement of the single heat sink beam due to the external force (P) at the central shuttle can be expressed as:

(27) $$v^{P}=\frac{PL^{3}}{12EI}$$

Considering the heat dissipation mechanism consisting of n number of heat sink beams, the total stiffness will be:

(28) $$K_{HS}=n\frac{12EI_{HS}}{L_{HS}{}^{3}}$$

To achieve the intended motion of the links of the microgripper, and to operate the microgripper safely, stiffness analysis of the overall structure is crucial. Therefore, stiffness analysis of the main structures (microgripper, heat dissipation mechanism, and thermal actuator) is performed.

Using Equations (24), (28) and (A23) in the Appendix A, and the parameters reported in Table 1, the stiffness of microgripper, thermal actuator, and heat dissipation mechanism are 14, 1728, and 89 μN/μm, respectively.

Based on the above results, the stiffness of the electrothermal actuator is considerably larger than the stiffness of the microgripper and the stiffness of the heat sink beams; therefore, the actuator can drive the microgripper properly.

### 2.4. Comparison between the Performances of the Microgripper, the Electrothermal Actuator, and the Heat Dissipation Mechanism Obtained from the Analytical Modeling and Simulations

To verify the validity of the theoretical modeling approach discussed in the previous sections, FEA is performed in ANSYS^TM^ multi-physics (2021 R1, American company based in Canonsburg, USA). Three-dimensional structural and coupled electric-thermal-mechanical simulations are conducted. The microgripper material is silicon, as in typical MEMS devices, and its main properties, used as input for the FE analysis are listed in Table 1.

More details regarding the simulation works and sensitivity analysis of the capacitive readout were previously reported by the authors [13].

#### 2.4.1. Microgripper’s Deformation and Stress

The length of the links and all the other parameters defining the microgripper geometry were identified upon an optimization process, which was carried out to grant a compact microgripper design (i.e., footprint of 4.3 × 3.5${\;\mathrm{mm}}^{2}$) that is compliant with the microfabrication constraints (i.e., minimum feature size) and operational requirements (i.e., tweezer offset and temperature), as reported in the Introduction. This optimization process resulted in the following values: $I_{3}B$ = 523.9 µm, $I_{3}A$ = 614.2 µm, $L_{1}$ = 775.2 µm, and $L_{2}$ = 487.6 µm. If we consider Equation (11), the overall amplification ratio ($R_{tot}$) can be 2.71.

Regarding the FEA, we performed a static structural analysis, where we applied a displacement of ~39 µm at the input end (i.e., at the shuttle of the microgripper). This indeed allowed the achievement of an output displacement of 52.5 µm at each tweezer’s arm (i.e., a total of 105 µm output displacement). Ideally, this displacement is intended for cell manipulation, where a typical cell diameter can be in the order of 15–20 µm [41], by considering an offset between the tweezer’s arms of 120 µm at rest to enable safe positioning in the vicinity of a cell.

**Figure 7:**
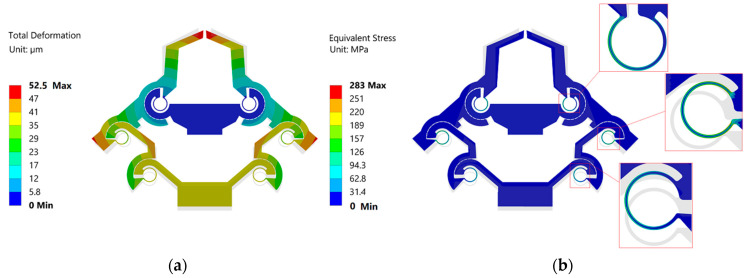
(**a**) Total displacement; (**b**) stress field when 39.16 µm input displacement is applied at the shuttle of the gripper.

The total displacement and stress field results obtained from the numerical simulations are shown in Figure 7a,b.

The output displacement of each tweezer resulted in a value of 52.5 µm (Figure 7a), i.e., the total output displacement of the tweezers was 105 µm, with a corresponding displacement amplification ratio of 2.68 (i.e., 105/39.16 µm). As shown in Figure 7b, the maximum stress of the microgripper was ~283 MPa, which is considerably less than the yield strength of the material (7 GPa); therefore, the device can be used safely.

By comparing the amplification ratio value obtained from the analytical modeling and simulations, there is a good match, with a relative difference of only ~1%, thus demonstrating the effectiveness of the PRBM approach in modeling the kinematics of the structure under investigation. The small difference between the analytical and numerical estimation can be due to different reasons, such as (i) the linkages are considered as rigid links in the theoretical model, but deformation occurred on the linkages in FEA simulation, and (ii) the rotation center of the flexure hinges drifted in FEA simulation, while in the theoretical modeling this cannot happen.

**Figure 8:**
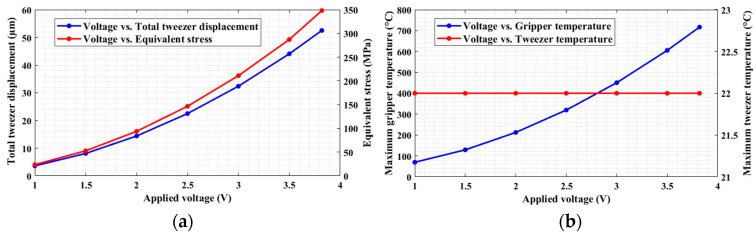
(**a**) Tweezer displacement and equivalent stress as functions of the applied voltage; (**b**) microgripper and tweezer temperature as functions of the applied voltage.

Figure 8 reports a plot showing the overall performance of the designed microgripper. In particular, it is possible to observe that the temperature in the gripper tweezer region is constant with 10 heat dissipation bars (which is around 22 °C) in a voltage range from 1 to 3.8 V, which is a safe temperature for biological sample manipulation. The range of the applied voltage is decided based on the desired output tweezer displacement, i.e., 52.5 µm. Moreover, the tweezer (jaw) and the overall gripper regions are considered for the analysis.

#### 2.4.2. Microgripper Stiffness

**Figure 9:**
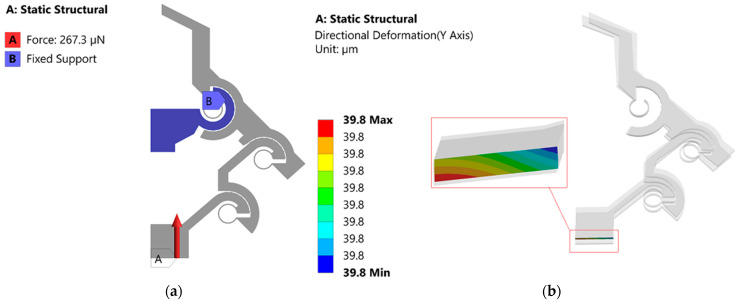
(**a**) Boundary conditions of the static structural analysis; (**b**) directional displacement along the vertical direction at the shuttle of the microgripper when a full range input displacement is applied.

Using Equation (24), the theoretical input stiffness of half of the microgripper is calculated as ~7 μN/μm, and regarding the FEA, we performed a static structural analysis, where we applied an input force (267.3 μN) at the input end (i.e., at the shuttle of the microgripper) (Figure 9a).

By considering an input force at the shuttle of the microgripper, the input stiffness of the half microgripper is (267.3/39.8) = 6.7 μN/μm. Therefore, the input stiffness’s obtained from analytical modeling and FEA have a good match with a relative difference of ~4.5%.

#### 2.4.3. Electrothermal Actuator

To verify Equation (A21) in the Appendix A, we performed a coupled steady-state thermal–static structural analysis, and we considered the same assumptions as the theoretical modeling, i.e., the central shuttle is rigid and not affected by the temperature increase. Geometrical parameters implemented in the numerical analysis are reported in Table 2.

**Figure 10:**
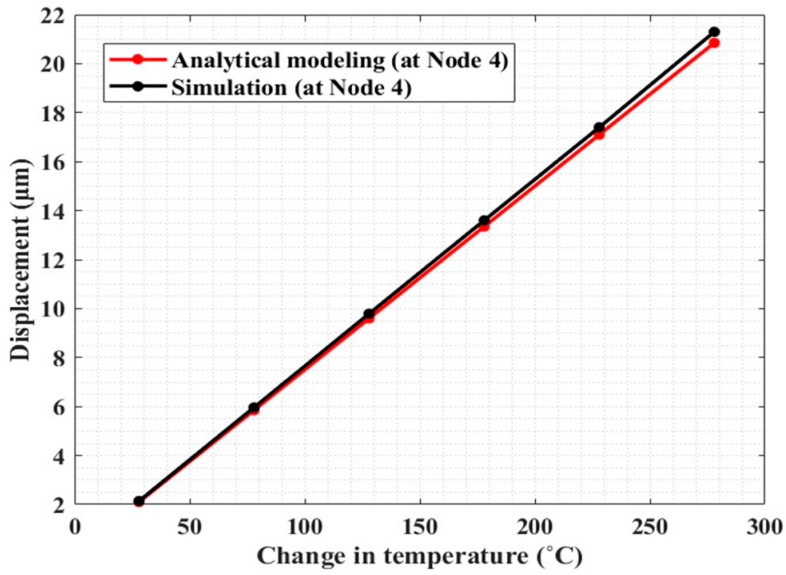
Comparison of the results, in terms of displacement vs. temperature change, obtained from analytical modeling and finite element simulations of the electrothermal actuator.

The displacements at node 4 obtained from analytical modeling and simulation have a good match with a relative difference of ~2.8% in temperature (ΔT) range from room temperature to 278 °C (Figure 10).

#### 2.4.4. Thermomechanical Actuation and Heat Dissipation Mechanism

Multi-physics analysis is also performed to provide an assessment of the temperature across the microgripper. The simulation is carried out by considering the full range input displacement of the gripper (~39 µm) to achieve the desired total output displacement of 105 µm. Moreover, we selected a beam angle of 2° by considering Figure 5a.

**Figure 11:**
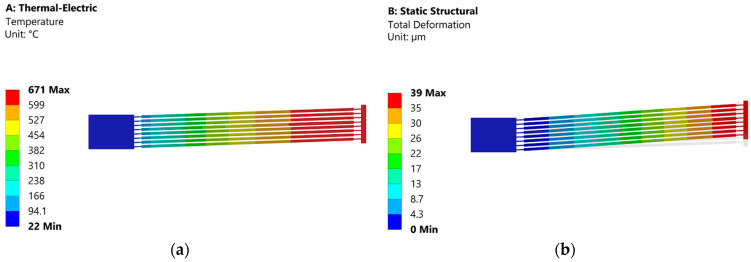
(**a**) Temperature field; (**b**) displacement field when the thermal actuator is biased with 6.2 V.

Figure 11b shows that we could achieve the intended 39 µm input displacement of the gripper with the proposed electrothermal model. However, the temperature at the tip of the actuator shuttle (Figure 11a) is significantly high.

To examine the effectiveness of heat sink beams in controlling the temperature increase around the tweezer region, the number of pairs of the heat sink beams is considered.

**Figure 12:**
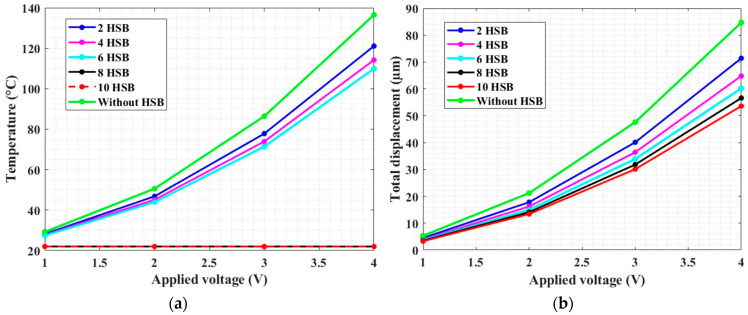
(**a**) Temperature at the tweezer region; (**b**) displacement at the tip of the tweezer for various numbers of heat sink beams (HSB) when the actuator is biased with 1–4 V.

Based on our intended input displacement and minimum temperature requirement around the tweezer region of the gripper, eight and ten numbers of heat sink beams can both be effective (Figure 12). However, we observed that there was an increment of temperature around the capacitive readout region with eight numbers of heat sink beams.

Indeed, for the safety of the microgripper structure, and to provide position feedback, rotary type capacitive sensors were implemented on the links of our microgripper. Therefore, changes in humidity or temperature can interfere with the operation of the sensors, and, in some cases, it can stop the sensor from working altogether [42].

By considering the drawbacks of temperature increase on the sensor, the electrothermal actuator with 10 pairs of heat sink beams is selected for our proposed microgripper structure.

## 3. Conclusions and Future Work

A conjugate-surface-flexure-hinge-based-microgripper is developed, which is capable of gripping biological cells of size ranging from 15 to 120 µm. In the tweezers region, ambient temperature (22 °C) is required to prevent the damage of biological samples due to high temperature. Based on fabrication technology limitations, a minimum feature size of 6 µm and a minimum gap size of 2 µm are considered during the design phase. A PRB equivalent model was developed for the proposed microgripper, for which an analytical model was developed from the electrothermal actuator with stepped beams. The model was used to optimize the actuator and the heat dissipation mechanism, providing an analytical model of the actuators with stepped actuation beams. Furthermore, simulations were carried out to verify the theoretical modeling. The displacements obtained from analytical modeling and simulation have a good match with a relative difference of ~2.8% in temperature (ΔT) range from room temperature to 278 °C.

The future work will focus on the fabrication of the proposed microgripper using standard MEMS technology from a silicon-on-insulator (SOI) wafer with deep reactive ion etching (DRIE) technique, and it will be packaged in a liquid proof housing to enable the first ever MEMS microgripper operation in liquid environment immersion, as required for in vitro manipulation of biological samples.

## Figures and Tables

**Table 1:** Silicon properties [40] and geometrical parameters.

Properties	Values	Properties	Values
Density	2330 kg/m^3^	Cross-Sectional Area of Beams $(A_{1}=A_{2}=A_{3}=A$)	$375\;{\mu m}^{2}$
Thermal expansion coefficient	$2.5\;\times \;10^{-6}\;\;{}^{\circ}C^{-1}$	Moment of inertia $(I_{1}=I_{2}=I_{3}=I$)	$7031.25\;{\mu m}^{4}$
Young’s modulus	130.1 GPa	Length of stepped beam element–1 $(L_{1}$)	40 µm
Poisson’s ratio	0.22	Length of stepped beam element–2 $(L_{2}$)	1150 µm
Thermal conductivity	148 W/(m ℃)	Length of stepped beam element–3 $(L_{3}$)	40 µm
Resistivity	0.005 Ω cm	Length of non-stepped beam $(l=L_{1}+L_{2}+L_{3}$)	1230 µm
Melting point	1415 ℃	Width of main beams $(w_{m}$)	15 µm
Reference temperature ($T_{0}$)	22 ℃	Width of short beams $(w_{s}$)	6 µm
Inclined beam angle (θ)	$2^{{}^{\circ}}$	Thickness of beams (t)	25 µm
Thickness, width, and radius of flexure hinge, respectively	25, 8, and 60 µm	Initial angle of flexure hinges ($\theta^{\prime }$)	$295^{{}^{\circ}}$
Total number of thermal actuator beams	16	Thickness, width, and length of heat sink beams, respectively	25, 10, and 900 µm
Total number of heat sink beams	20		

**Table 2:** Geometrical parameters of the thermal actuator.

Geometrical Parameters	Values
Total Number of Beams	16
Pre-bending angle of beams	$2^{{}^{\circ}}$
Length of main beams	1150 μm
Width of main beams	15 μm
Length of short beams	40 μm
Width of short beams	6 μm
Gap between beams	8 μm
Shuttle width	40 μm

## Supplementary Materials

The following supporting information can be downloaded at: https://www.mdpi.com/article/10.3390/mi13091391/s1, Video S1: Thermally actuated CSFH-based microgripper.

## Appendix A

1. Computation of the elastic stiffness matrix in a local reference frame (u′, v′), as shown in Figure 7.

(A1) $${\left[K_{e}\right]}_{local}\left\{d\right\}=\{f_{M}\}+\{f_{T}\}\;$$

where $\left[K_{e}\right]$ is the element stiffness matrix, {d} is the nodal displacement vector, $\{f_{M}\}$ is the mechanical nodal force, and $\{f_{T}\}$ is the thermal force.

The thermal nodal forces can be computed as [40]:

(A2) $$\{f_{T}\}=\left\{\begin{array}{c} f_{T_{i}}\\ f_{T_{i+1}} \end{array}\right\}=\left\{\begin{array}{c} -\alpha \Delta TEA_{i}\\ \alpha \Delta TEA_{i} \end{array}\right\}i=1,\;2,\;and\;3$$

Element–1

(A3) $$\left[\begin{array}{c} \frac{EA_{1}}{L_{1}}\\ 0\\ 0\\ -\frac{EA_{1}}{L_{1}} \end{array}\;\;\;\begin{array}{c} 0\\ \frac{12EI_{1}}{L_{1}^{3}}\\ \frac{6EI_{1}}{L_{1}^{2}}\\ 0 \end{array}\;\;\;\begin{array}{c} 0\\ \frac{6EI_{1}}{L_{1}^{2}}\\ \frac{4EI_{1}}{L_{1}}\\ 0 \end{array}\;\;\;\begin{array}{c} -\frac{EA_{1}}{L_{1}}\\ 0\\ 0\\ \frac{EA_{1}}{L_{1}} \end{array}\right]\left\{\begin{array}{c} {u^{\prime }}_{1}\\ {v^{\prime }}_{1}\\ {u^{\prime }}_{2}\\ {v^{\prime }}_{2} \end{array}\right\}=\left\{\begin{array}{c} -\alpha \Delta TEA_{1}\\ 0\\ \alpha \Delta TEA_{1}\\ 0 \end{array}\right\}+\left\{\begin{array}{c} R_{u}^{1^{\prime }}\\ 0\\ 0\\ 0 \end{array}\right\}$$

Element–2

(A4) $$\left[\begin{array}{c} \frac{EA_{2}}{L_{2}}\\ 0\\ 0\\ -\frac{EA_{2}}{L_{2}} \end{array}\;\;\;\begin{array}{c} 0\\ \frac{12EI_{2}}{L_{2}^{3}}\\ \frac{6EI_{2}}{L_{2}^{2}}\\ 0 \end{array}\;\;\;\begin{array}{c} 0\\ \frac{6EI_{2}}{L_{2}^{2}}\\ \frac{4EI_{2}}{L_{2}}\\ 0 \end{array}\;\;\;\begin{array}{c} -\frac{EA_{2}}{L_{2}}\\ 0\\ 0\\ \frac{EA_{2}}{L_{2}} \end{array}\right]\left\{\begin{array}{c} {u^{\prime }}_{2}\\ {v^{\prime }}_{2}\\ {u^{\prime }}_{3}\\ {v^{\prime }}_{3} \end{array}\right\}=\left\{\begin{array}{c} -\alpha \Delta TEA_{2}\\ 0\\ \alpha \Delta TEA_{2}\\ 0 \end{array}\right\}$$

Element–3

(A5) $$\left[\begin{array}{c} \frac{EA_{3}}{L_{3}}\\ 0\\ 0\\ -\frac{EA_{3}}{L_{3}} \end{array}\;\;\;\begin{array}{c} 0\\ \frac{12EI_{3}}{L_{3}^{3}}\\ \frac{6EI_{3}}{L_{3}^{2}}\\ 0 \end{array}\;\;\;\begin{array}{c} 0\\ \frac{6EI_{2}}{L_{2}^{2}}\\ \frac{4EI_{2}}{L_{3}}\\ 0 \end{array}\;\;\;\begin{array}{c} -\frac{EA_{3}}{L_{3}}\\ 0\\ 0\\ \frac{EA_{3}}{L_{3}} \end{array}\right]\left\{\begin{array}{c} {u^{\prime }}_{3}\\ {v^{\prime }}_{3}\\ {u^{\prime }}_{4}\\ {v^{\prime }}_{4} \end{array}\right\}=\left\{\begin{array}{c} -\alpha \Delta TEA_{3}\\ 0\\ \alpha \Delta TEA_{3}\\ 0 \end{array}\right\}+\left\{\begin{array}{c} 0\\ 0\\ R_{u}^{4^{\prime }}\\ 0 \end{array}\right\}$$

2. Transformation of the local stiffness matrix to global stiffness matrix by means of a rotation matrix.

Relation between local and global stiffness:

(A6) $$\left[K\right]{\;}_{global}={\left[T\right]}^{T}{\left[K\right]}_{local}\left[T\right]$$

By considering the axial stiffness:

(A7) $$\left[K\right]{\;}_{global-1}=\frac{EA}{L}\left[\begin{array}{c} c^{2}\\ cs\\ -c^{2}\\ -cs \end{array}\;\;\;\begin{array}{c} cs\\ s^{2}\\ -cs\\ -s^{2} \end{array}\;\;\;\begin{array}{c} -c^{2}\\ -cs\\ c^{2}\\ cs \end{array}\;\;\;\begin{array}{c} -cs\\ -s^{2}\\ cs\\ s^{2} \end{array}\right]$$

By considering the bending stiffness:

(A8) $$\left[K\right]{\;}_{global-2}=\frac{EI}{L^{3}}\left[\begin{array}{c} 12s^{2}\\ -12cs\\ -12s^{2}\\ 12cs \end{array}\;\;\;\begin{array}{c} -12cs\\ 12c^{2}\\ 12cs\\ -12c^{2} \end{array}\;\;\;\begin{array}{c} -12s^{2}\\ 12cs\\ 12s^{2}\\ -12cs \end{array}\;\;\;\begin{array}{c} 12cs\\ -12c^{2}\\ -12cs\\ 12c^{2} \end{array}\right]$$

Therefore,

(A9) $$\left[K\right]{\;}_{global}=\left[K\right]{\;}_{global-1}+\left[K\right]{\;}_{global-2}$$

Relation between local and global force vectors:

(A10) $${\left\{f\right\}}_{global}=\left[T\right]{\left\{f\right\}}_{local}$$

where [T]= $\left[\begin{array}{c} c\\ s\\ 0\\ 0 \end{array}\;\begin{array}{c} -s\\ c\\ 0\\ 0 \end{array}\;\begin{array}{c} 0\\ 0\\ c\\ s \end{array}\;\begin{array}{c} 0\\ 0\\ -s\\ c \end{array}\right]$

By combining Equations (A6)–(A10), Equations (A3)–(A5) transform to:

Element–1

(A11) $$\left[\begin{array}{cccc} \frac{EA_{1}}{L_{1}}c^{2}+\frac{12EI_{1}}{L_{1}^{3}}s^{2} & \frac{EA_{1}}{L_{1}}cs-\frac{12EI_{1}}{L_{1}^{3}}cs & -\frac{EA_{1}}{L_{1}}c^{2}-\frac{12EI_{1}}{L_{1}^{3}}s^{2} & -\frac{EA_{1}}{L_{1}}cs+\frac{12EI_{1}}{L_{1}^{3}}cs\\ \frac{EA_{1}}{L_{1}}cs-\frac{12EI_{1}}{L_{1}^{3}}cs & \frac{EA_{1}}{L_{1}}s^{2}+\frac{12EI_{1}}{L_{1}^{3}}c^{2} & -\frac{EA_{1}}{L_{1}}cs+\frac{12EI_{1}}{L_{1}^{3}}cs & -\frac{EA_{1}}{L_{1}}s^{2}-\frac{12EI_{1}}{L_{1}^{3}}c^{2}\\ -\frac{EA_{1}}{L_{1}}c^{2}-\frac{12EI_{1}}{L_{1}^{3}}s^{2} & -\frac{EA_{1}}{L_{1}}cs+\frac{12EI_{1}}{L_{1}^{3}}cs & \frac{EA_{1}}{L_{1}}c^{2}+\frac{12EI_{1}}{L_{1}^{3}}s^{2} & \frac{EA_{1}}{L_{1}}cs-\frac{12EI_{1}}{L_{1}^{3}}cs\\ -\frac{EA_{1}}{L_{1}}cs+\frac{12EI_{1}}{L_{1}^{3}}cs & -\frac{EA_{1}}{L_{1}}s^{2}-\frac{12EI_{1}}{L_{1}^{3}}c^{2} & \frac{EA_{1}}{L_{1}}cs-\frac{12EI_{1}}{L_{1}^{3}}cs & \frac{EA_{1}}{L_{1}}s^{2}+\frac{12EI_{1}}{L_{1}^{3}}c^{2} \end{array}\right]\left\{\begin{array}{c} u_{1}\\ v_{1}\\ u_{2}\\ v_{2} \end{array}\right\}=\;\left\{\begin{array}{c} -\alpha \Delta TEA_{1}c\\ -\alpha \Delta TEA_{1}s\\ \alpha \Delta TEA_{1}c\\ \alpha \Delta TEA_{1}s \end{array}\right\}+\left\{\begin{array}{c} R_{u}^{1}\\ 0\\ 0\\ 0 \end{array}\right\}$$

Element–2

(A12) $$\left[\begin{array}{cccc} \frac{EA_{2}}{L_{2}}c^{2}+\frac{12EI_{2}}{L_{2}^{3}}s^{2} & \frac{EA_{2}}{L_{2}}cs-\frac{12EI_{2}}{L_{2}^{3}}cs & -\frac{EA_{2}}{L_{2}}c^{2}-\frac{12EI_{2}}{L_{2}^{3}}s^{2} & -\frac{EA_{2}}{L_{2}}cs+\frac{12EI_{2}}{L_{2}^{3}}cs\\ \frac{EA_{2}}{L_{2}}cs-\frac{12EI_{2}}{L_{2}^{3}}cs & \frac{EA_{2}}{L_{2}}s^{2}+\frac{12EI_{2}}{L_{2}^{3}}c^{2} & -\frac{EA_{2}}{L_{2}}cs+\frac{12EI_{2}}{L_{2}^{3}}cs & -\frac{EA_{2}}{L_{2}}s^{2}-\frac{12EI_{2}}{L_{2}^{3}}c^{2}\\ -\frac{EA_{2}}{L_{2}}c^{2}-\frac{12EI_{2}}{L_{2}^{3}}s^{2} & -\frac{EA_{2}}{L_{2}}cs+\frac{12EI_{2}}{L_{2}^{3}}cs & \frac{EA_{2}}{L_{2}}c^{2}+\frac{12EI_{2}}{L_{2}^{3}}s^{2} & \frac{EA_{2}}{L_{2}}cs-\frac{12EI_{2}}{L_{2}^{3}}cs\\ -\frac{EA_{2}}{L_{2}}cs+\frac{12EI_{2}}{L_{2}^{3}}cs & -\frac{EA_{2}}{L_{2}}s^{2}-\frac{12EI_{2}}{L_{2}^{3}}c^{2} & \frac{EA_{2}}{L_{2}}cs-\frac{12EI_{2}}{L_{2}^{3}}cs & \frac{EA_{2}}{L_{2}}s^{2}+\frac{12EI_{2}}{L_{2}^{3}}c^{2} \end{array}\right]\left\{\begin{array}{c} u_{2}\\ v_{2}\\ u_{3}\\ v_{3} \end{array}\right\}=\;\left\{\begin{array}{c} -\alpha \Delta TEA_{2}c\\ -\alpha \Delta TEA_{2}s\\ \alpha \Delta TEA_{2}c\\ \alpha \Delta TEA_{2}s \end{array}\right\}$$

Element–3

(A13) $$\left[\begin{array}{cccc} \frac{EA_{3}}{L_{3}}c^{2}+\frac{12EI_{3}}{L_{3}^{3}}s^{2} & \frac{EA_{3}}{L_{3}}cs-\frac{12EI_{3}}{L_{3}^{3}}cs & -\frac{EA_{3}}{L_{3}}c^{2}-\frac{12EI_{3}}{L_{3}^{3}}s^{2} & -\frac{EA_{3}}{L_{3}}cs+\frac{12EI_{3}}{L_{1}^{3}}cs\\ \frac{EA_{3}}{L_{3}}cs-\frac{12EI_{3}}{L_{3}^{3}}cs & \frac{EA_{3}}{L_{3}}s^{2}+\frac{12EI_{3}}{L_{3}^{3}}c^{2} & -\frac{EA_{3}}{L_{3}}cs+\frac{12EI_{3}}{L_{3}^{3}}cs & -\frac{EA_{3}}{L_{3}}s^{2}-\frac{12EI_{3}}{L_{3}^{3}}c^{2}\\ -\frac{EA_{3}}{L_{3}}c^{2}-\frac{12EI_{3}}{L_{3}^{3}}s^{2} & -\frac{EA_{3}}{L_{3}}cs+\frac{12EI_{3}}{L_{3}^{3}}cs & \frac{EA_{3}}{L_{3}}c^{2}+\frac{12EI_{3}}{L_{3}^{3}}s^{2} & \frac{EA_{3}}{L_{3}}cs-\frac{12EI_{3}}{L_{3}^{3}}cs\\ -\frac{EA_{3}}{L_{3}}cs+\frac{12EI_{3}}{L_{3}^{3}}cs & -\frac{EA_{3}}{L_{3}}s^{2}-\frac{12EI_{3}}{L_{3}^{3}}c^{2} & \frac{EA_{3}}{L_{3}}cs-\frac{12EI_{3}}{L_{3}^{3}}cs & \frac{EA_{3}}{L_{3}}s^{2}+\frac{12EI_{3}}{L_{3}^{3}}c^{2} \end{array}\right].\left\{\begin{array}{c} u_{3}\\ v_{3}\\ u_{4}\\ v_{4} \end{array}\right\}=\;\left\{\begin{array}{c} -\alpha \Delta TEA_{3}c\\ -\alpha \Delta TEA_{3}s\\ \alpha \Delta TEA_{3}c\\ \alpha \Delta TEA_{3}s \end{array}\right\}+\left\{\begin{array}{c} 0\\ 0\\ R_{u}^{4}\\ 0 \end{array}\right\}$$

3. Assembly of the element matrices.

(A14)
$$\left[\begin{array}{cccccccc} \frac{EA_{1}}{L_{1}}c^{2}+\frac{12EI_{1}}{L_{1}^{3}}s^{2} & \frac{EA_{1}}{L_{1}}cs-\frac{12EI_{1}}{L_{1}^{3}}cs & -\frac{EA_{1}}{L_{1}}c^{2}-\frac{12EI_{1}}{L_{1}^{3}}s^{2} & -\frac{EA_{1}}{L_{1}}cs+\frac{12EI_{1}}{L_{1}^{3}}cs & 0 & 0 & 0 & 0\\ \frac{EA_{1}}{L_{1}}cs-\frac{12EI_{1}}{L_{1}^{3}}cs & \frac{EA_{1}}{L_{1}}s^{2}+\frac{12EI_{1}}{L_{1}^{3}}c^{2} & -\frac{EA_{1}}{L_{1}}cs+\frac{12EI_{1}}{L_{1}^{3}}cs & -\frac{EA_{1}}{L_{1}}s^{2}-\frac{12EI_{1}}{L_{1}^{3}}c^{2} & 0 & 0 & 0 & 0\\ -\frac{EA_{1}}{L_{1}}c^{2}-\frac{12EI_{1}}{L_{1}^{3}}s^{2} & -\frac{EA_{1}}{L_{1}}cs+\frac{12EI_{1}}{L_{1}^{3}}cs & \left(\frac{EA_{1}}{L_{1}}c^{2}+\frac{12EI_{1}}{L_{1}^{3}}s^{2}\right)+\left(\frac{EA_{2}}{L_{2}}c^{2}+\frac{12EI_{2}}{L_{2}^{3}}s^{2}\right) & \left(\frac{EA_{1}}{L_{1}}cs-\frac{12EI_{1}}{L_{1}^{3}}cs\right)+\left(\frac{EA_{2}}{L_{2}}cs-\frac{12EI_{2}}{L_{2}^{3}}cs\right) & -\frac{EA_{2}}{L_{2}}c^{2}-\frac{12EI_{2}}{L_{2}^{3}}s^{2} & -\frac{EA_{2}}{L_{2}}cs+\frac{12EI_{2}}{L_{2}^{3}}cs & 0 & 0\\ -\frac{EA_{1}}{L_{1}}cs+\frac{12EI_{1}}{L_{1}^{3}}cs & -\frac{EA_{1}}{L_{1}}s^{2}-\frac{12EI_{1}}{L_{1}^{3}}c^{2} & \left(\frac{EA_{1}}{L_{1}}cs-\frac{12EI_{1}}{L_{1}^{3}}cs\right)+\left(\frac{EA_{2}}{L_{2}}cs-\frac{12EI_{2}}{L_{2}^{3}}cs\right) & \left(\frac{EA_{1}}{L_{1}}s^{2}+\frac{12EI_{1}}{L_{1}^{3}}c^{2}\right)+\;(\frac{EA_{2}}{L_{2}}s^{2}+\frac{12EI_{2}}{L_{2}^{3}}c^{2}) & -\frac{EA_{2}}{L_{2}}cs+\frac{12EI_{2}}{L_{2}^{3}}cs & -\frac{EA_{2}}{L_{2}}s^{2}-\frac{12EI_{2}}{L_{2}^{3}}c^{2} & 0 & 0\\ 0 & 0 & -\frac{EA_{2}}{L_{2}}c^{2}-\frac{6EI_{2}}{L_{2}^{3}}s^{2} & -\frac{EA_{2}}{L_{2}}cs+\frac{12EI_{2}}{L_{2}^{3}}cs & \frac{EA_{2}}{L_{2}}c^{2}+\frac{12EI_{2}}{L_{2}^{3}}s^{2}+\;\frac{EA_{3}}{L_{3}}c^{2}+\frac{12EI_{3}}{L_{3}^{3}}s^{2} & \frac{EA_{2}}{L_{2}}cs-\frac{12EI_{2}}{L_{2}^{3}}cs+\;\frac{EA_{3}}{L_{3}}cs-\frac{12EI_{3}}{L_{3}^{3}}cs & -\frac{EA_{3}}{L_{3}}c^{2}-\frac{12EI_{3}}{L_{3}^{3}}s^{2} & -\frac{EA_{3}}{L_{3}}cs+\frac{12EI_{3}}{L_{3}^{3}}cs\\ 0 & 0 & -\frac{EA_{2}}{L_{2}}cs+\frac{12EI_{2}}{L_{2}^{3}}cs & -\frac{EA_{2}}{L_{2}}s^{2}-\frac{12EI_{2}}{L_{2}^{3}}c^{2} & \frac{EA_{2}}{L_{2}}cs-\frac{12EI_{2}}{L_{2}^{3}}cs+\;\frac{EA_{3}}{L_{3}}cs-\frac{12EI_{3}}{L_{3}^{3}}cs & \frac{EA_{2}}{L_{2}}s^{2}+\frac{12EI_{2}}{L_{2}^{3}}c^{2}+\;\frac{EA_{3}}{L_{3}}s^{2}+\frac{12EI_{3}}{L_{3}^{3}}c^{2} & -\frac{EA_{3}}{L_{3}}cs+\frac{12EI_{3}}{L_{3}^{3}}cs & -\frac{EA_{3}}{L_{3}}s^{2}-\frac{12EI_{3}}{L_{3}^{3}}c^{2}\\ 0 & 0 & 0 & 0 & -\frac{EA_{3}}{L_{3}}c^{2}-\frac{12EI_{3}}{L_{3}^{3}}s^{2} & -\frac{EA_{3}}{L_{3}}cs+\frac{12EI_{3}}{L_{3}^{3}}cs & \frac{EA_{3}}{L_{3}}c^{2}+\frac{12EI_{3}}{L_{3}^{3}}s^{2} & \frac{EA_{3}}{L_{3}}cs-\frac{12EI_{3}}{L_{3}^{3}}cs\\ 0 & 0 & 0 & 0 & -\frac{EA_{3}}{L_{3}}cs+\frac{12EI_{3}}{L_{3}^{3}}cs & -\frac{EA_{3}}{L_{3}}s^{2}-\frac{12EI_{3}}{L_{3}^{3}}c^{2} & \frac{EA_{3}}{L_{3}}cs-\frac{12EI_{3}}{L_{3}^{3}}cs & \frac{EA_{3}}{L_{3}}s^{2}+\frac{12EI_{3}}{L_{3}^{3}}c^{2} \end{array}\right]\left\{\begin{array}{c} \begin{array}{c} \begin{array}{c} u_{1}\\ v_{1}\\ u_{2}\\ v_{2} \end{array}\\ u_{3} \end{array}\\ v_{3}\\ u_{4}\\ v_{4} \end{array}\right\}=\left\{\begin{array}{c} -\alpha \Delta TEA_{1}c\\ -\alpha \Delta TEA_{1}s\\ \alpha \Delta TEA_{1}c-\alpha \Delta TEA_{2}c\\ \alpha \Delta TEA_{1}s-\alpha \Delta TEA_{2}s\\ \begin{array}{c} \alpha \Delta TEA_{2}c-\alpha \Delta TEA_{3}c\\ \alpha \Delta TEA_{2}s-\alpha \Delta TEA_{3}s \end{array}\\ \begin{array}{c} \alpha \Delta TEA_{3}c\\ \alpha \Delta TEA_{3}s \end{array} \end{array}\right\}+\left\{\begin{array}{c} R_{u}^{1}\\ 0\\ 0\\ 0\\ 0\\ 0\\ R_{u}^{4}\\ 0 \end{array}\right\}\;$$

4. Boundary conditions.

$$u_{1}=u_{4}=0,$$

$$v_{1}=0,$$

5. Imposition of boundary conditions in the global matrix (A14) to find the displacement at node 4, and the global matrix is simplified as follows:

(A15)
$$\left[\begin{array}{cccccccc} \frac{EA_{1}}{L_{1}}c^{2}+\frac{12EI_{1}}{L_{1}^{3}}s^{2} & \frac{EA_{1}}{L_{1}}cs-\frac{12EI_{1}}{L_{1}^{3}}cs & -\frac{EA_{1}}{L_{1}}c^{2}-\frac{12EI_{1}}{L_{1}^{3}}s^{2} & -\frac{EA_{1}}{L_{1}}cs+\frac{12EI_{1}}{L_{1}^{3}}cs & 0 & 0 & 0 & 0\\ \frac{EA_{1}}{L_{1}}cs-\frac{12EI_{1}}{L_{1}^{3}}cs & \frac{EA_{1}}{L_{1}}s^{2}+\frac{12EI_{1}}{L_{1}^{3}}c^{2} & -\frac{EA_{1}}{L_{1}}cs+\frac{12EI_{1}}{L_{1}^{3}}cs & -\frac{EA_{1}}{L_{1}}s^{2}-\frac{12EI_{1}}{L_{1}^{3}}c^{2} & 0 & 0 & 0 & 0\\ -\frac{EA_{1}}{L_{1}}c^{2}-\frac{12EI_{1}}{L_{1}^{3}}s^{2} & -\frac{EA_{1}}{L_{1}}cs+\frac{12EI_{1}}{L_{1}^{3}}cs & \left(\frac{EA_{1}}{L_{1}}c^{2}+\frac{12EI_{1}}{L_{1}^{3}}s^{2}\right)+\left(\frac{EA_{2}}{L_{2}}c^{2}+\frac{12EI_{2}}{L_{2}^{3}}s^{2}\right) & \left(\frac{EA_{1}}{L_{1}}cs-\frac{12EI_{1}}{L_{1}^{3}}cs\right)+\left(\frac{EA_{2}}{L_{2}}cs-\frac{12EI_{2}}{L_{2}^{3}}cs\right) & -\frac{EA_{2}}{L_{2}}c^{2}-\frac{12EI_{2}}{L_{2}^{3}}s^{2} & -\frac{EA_{2}}{L_{2}}cs+\frac{12EI_{2}}{L_{2}^{3}}cs & 0 & 0\\ -\frac{EA_{1}}{L_{1}}cs+\frac{12EI_{1}}{L_{1}^{3}}cs & -\frac{EA_{1}}{L_{1}}s^{2}-\frac{12EI_{1}}{L_{1}^{3}}c^{2} & \left(\frac{EA_{1}}{L_{1}}cs-\frac{12EI_{1}}{L_{1}^{3}}cs\right)+\left(\frac{EA_{2}}{L_{2}}cs-\frac{12EI_{2}}{L_{2}^{3}}cs\right) & \left(\frac{EA_{1}}{L_{1}}s^{2}+\frac{12EI_{1}}{L_{1}^{3}}c^{2}\right)+\;(\frac{EA_{2}}{L_{2}}s^{2}+\frac{12EI_{2}}{L_{2}^{3}}c^{2}) & -\frac{EA_{2}}{L_{2}}cs+\frac{12EI_{2}}{L_{2}^{3}}cs & -\frac{EA_{2}}{L_{2}}s^{2}-\frac{12EI_{2}}{L_{2}^{3}}c^{2} & 0 & 0\\ 0 & 0 & -\frac{EA_{2}}{L_{2}}c^{2}-\frac{12EI_{2}}{L_{2}^{3}}s^{2} & -\frac{EA_{2}}{L_{2}}cs+\frac{12EI_{2}}{L_{2}^{3}}cs & \left(\frac{EA_{2}}{L_{2}}c^{2}+\frac{12EI_{2}}{L_{2}^{3}}s^{2})+\;(\frac{EA_{3}}{L_{3}}c^{2}+\frac{12EI_{3}}{L_{3}^{3}}s^{2}\right) & \left(\frac{EA_{2}}{L_{2}}cs-\frac{12EI_{2}}{L_{2}^{3}}cs)+\;(\frac{EA_{3}}{L_{3}}cs-\frac{12EI_{3}}{L_{3}^{3}}cs\right) & -\frac{EA_{3}}{L_{3}}c^{2}-\frac{12EI_{3}}{L_{3}^{3}}s^{2} & -\frac{EA_{3}}{L_{3}}cs+\frac{12EI_{3}}{L_{3}^{3}}cs\\ 0 & 0 & -\frac{EA_{2}}{L_{2}}cs+\frac{12EI_{2}}{L_{2}^{3}}cs & -\frac{EA_{2}}{L_{2}}s^{2}-\frac{12EI_{2}}{L_{2}^{3}}c^{2} & \left(\frac{EA_{2}}{L_{2}}cs-\frac{12EI_{2}}{L_{2}^{3}}cs)+\;(\frac{EA_{3}}{L_{3}}cs-\frac{12EI_{3}}{L_{3}^{3}}cs\right) & \left(\frac{EA_{2}}{L_{2}}s^{2}+\frac{12EI_{2}}{L_{2}^{3}}c^{2})+\;(\frac{EA_{3}}{L_{3}}s^{2}+\frac{12EI_{3}}{L_{3}^{3}}c^{2}\right) & -\frac{EA_{3}}{L_{3}}cs+\frac{12EI_{3}}{L_{3}^{3}}cs & -\frac{EA_{3}}{L_{3}}s^{2}-\frac{12EI_{3}}{L_{3}^{3}}c^{2}\\ 0 & 0 & 0 & 0 & -\frac{EA_{3}}{L_{3}}c^{2}-\frac{12EI_{3}}{L_{3}^{3}}s^{2} & -\frac{EA_{3}}{L_{3}}cs+\frac{12EI_{3}}{L_{3}^{3}}cs & \frac{EA_{3}}{L_{3}}c^{2}+\frac{12EI_{3}}{L_{3}^{3}}s^{2} & \frac{EA_{3}}{L_{3}}cs-\frac{12EI_{3}}{L_{3}^{3}}cs\\ 0 & 0 & 0 & 0 & -\frac{EA_{3}}{L_{3}}cs+\frac{12EI_{3}}{L_{3}^{3}}cs & -\frac{EA_{3}}{L_{3}}s^{2}-\frac{12EI_{3}}{L_{3}^{3}}c^{2} & \frac{EA_{3}}{L_{3}}cs-\frac{12EI_{3}}{L_{3}^{3}}cs & \frac{EA_{3}}{L_{3}}s^{2}+\frac{12EI_{3}}{L_{3}^{3}}c^{2} \end{array}\right]\left\{\begin{array}{c} u_{1}^{\Delta T}=0\\ v_{1}^{\Delta T}=0\\ u_{2}^{\Delta T}\\ v_{2}^{\Delta T}\;\\ u_{3}^{\Delta T}\\ v_{3}^{\Delta T}\\ u_{4}^{\Delta T}=0\\ v_{4}^{\Delta T}\; \end{array}\right\}=\left\{\begin{array}{c} -\alpha \Delta TEA_{1}c\\ -\alpha \Delta TEA_{1}s\\ \alpha \Delta TEA_{1}c-\alpha \Delta TEA_{2}c\\ \alpha \Delta TEA_{1}s-\alpha \Delta TEA_{2}s\\ \alpha \Delta TEA_{2}c-\alpha \Delta TEA_{3}c\\ \alpha \Delta TEA_{2}s-\alpha \Delta TEA_{3}s\\ \alpha \Delta TEA_{3}c\\ \alpha \Delta TEA_{3}s \end{array}\right\}\;$$

After imposing the boundary conditions in the assembly matrix (A14), we achieved five simultaneous equations (i.e., obtained from removing the rows and columns corresponding to the variables equal to zero), and for the purpose of simplicity, we assigned the coefficients with a symbol as follows:

(A16a) $$\begin{array}{l} u_{2}^{\Delta T}\left[\left(\frac{EA_{1}}{L_{1}}c^{2}+\frac{12EI_{1}}{L_{1}^{3}}s^{2}\right)+\left(\frac{EA_{2}}{L_{2}}c^{2}+\frac{12EI_{2}}{L_{2}^{3}}s^{2}\right)\right]+v_{2}^{\Delta T}\left[\left(\frac{EA_{1}}{L_{1}}cs-\frac{12EI_{1}}{L_{1}^{3}}cs\right)+\left(\frac{EA_{2}}{L_{2}}cs-\frac{12EI_{2}}{L_{2}^{3}}cs\right)\right]+\\ u_{3}^{\Delta T}\left(-\frac{EA_{2}}{L_{2}}c^{2}-\frac{12EI_{2}}{L_{2}^{3}}s^{2}\right)+v_{3}^{\Delta T}\left(-\frac{EA_{2}}{L_{2}}cs+\frac{12EI_{2}}{L_{2}^{3}}cs\right)=\alpha \Delta TEA_{1}c-\alpha \Delta TEA_{2}c \end{array}$$

(A16b)
$$u_{2}^{\Delta T}\left(I+N\right)+v_{2}^{\Delta T}\left(F+K\right)+u_{3}^{\Delta T}\left(O\right)+v_{3}^{\Delta T}\left(L\right)=\alpha \Delta TEA_{1}c-\alpha \Delta TEA_{2}c$$

(A17a)
$$\begin{array}{l} u_{2}^{\Delta T}\left[\left(\frac{EA_{1}}{L_{1}}cs-\frac{12EI_{1}}{L_{1}^{3}}cs\right)+\left(\frac{EA_{2}}{L_{2}}cs-\frac{12EI_{2}}{L_{2}^{3}}cs\right)\right]+v_{2}^{\Delta T}\left[\left(\frac{EA_{1}}{L_{1}}s^{2}+\frac{12EI_{1}}{L_{1}^{3}}c^{2}\right)+(\frac{EA_{2}}{L_{2}}s^{2}+\frac{12EI_{2}}{L_{2}^{3}}c^{2})\right]+\\ u_{3}^{\Delta T}\left(-\frac{EA_{2}}{L_{2}}cs+\frac{12EI_{2}}{L_{2}^{3}}cs\right)\;+v_{3}^{\Delta T}\left(-\frac{EA_{2}}{L_{2}}s^{2}-\frac{12EI_{2}}{L_{2}^{3}}c^{2}\right)=\alpha \Delta {\mathrm{TEA}}_{1}s\;-\;\alpha \Delta {\mathrm{TEA}}_{2}s \end{array}$$

(A17b)
$$u_{2}^{\Delta T}\left(F+K\right)+v_{2}^{\Delta T}\left(H+M\right)+u_{3}^{\Delta T}\left(L\right)\;+v_{3}^{\Delta T}\left(R\right)=\alpha \Delta {\mathrm{TEA}}_{1}s\;-\;\alpha \Delta TEA_{2}s$$

(A18a)
$$\begin{array}{l} u_{2}^{\Delta T}\left(-\frac{EA_{2}}{L_{2}}c^{2}-\frac{12EI_{2}}{L_{2}^{3}}s^{2}\right)+v_{2}^{\Delta T}\left(-\frac{EA_{2}}{L_{2}}cs+\frac{12EI_{2}}{L_{2}^{3}}cs\right)+u_{3}^{\Delta T}\left[\left(\frac{EA_{2}}{L_{2}}c^{2}+\frac{12EI_{2}}{L_{2}^{3}}s^{2}\right)+\left(\frac{EA_{3}}{L_{3}}c^{2}+\frac{12EI_{3}}{L_{3}^{3}}s^{2}\right)\right]+\\ v_{3}^{\Delta T}\left[\left(\frac{EA_{2}}{L_{2}}cs-\frac{12EI_{2}}{L_{2}^{3}}cs\right)+\left(\frac{EA_{3}}{L_{3}}cs-\frac{12EI_{3}}{L_{3}^{3}}cs\right)\right]+v_{4}^{\Delta T}\left(-\frac{EA_{3}}{L_{3}}cs+\frac{12EI_{3}}{L_{3}^{3}}cs\right)=\alpha \Delta TEA_{2}c-\alpha \Delta TEA_{3}c \end{array}$$

(A18b)
$$u_{2}^{\Delta T}\left(O\right)+v_{2}^{\Delta T}\left(L\right)+u_{3}^{\Delta T}\left[N+D\right]+v_{3}^{\Delta T}\left[K+B\right]+v_{4}^{\Delta T}\left(A\right)=\alpha \Delta TEA_{2}c-\alpha \Delta TEA_{3}c$$

(A19a)
$$\begin{array}{l} u_{2}^{\Delta T}\left(-\frac{EA_{2}}{L_{2}}cs+\frac{12EI_{2}}{L_{2}^{3}}cs\right)+v_{2}^{\Delta T}\left(-\frac{EA_{2}}{L_{2}}s^{2}-\frac{12EI_{2}}{L_{2}^{3}}c^{2}\right)+u_{3}^{\Delta T}\left[\left(\frac{EA_{2}}{L_{2}}cs-\frac{12EI_{2}}{L_{2}^{3}}cs\right)+\left(\frac{EA_{3}}{L_{3}}cs-\frac{12EI_{3}}{L_{3}^{3}}cs\right)\right]\;+\\ v_{3}^{\Delta T}\left[\left(\frac{EA_{2}}{L_{2}}s^{2}+\frac{12EI_{2}}{L_{2}^{3}}c^{2}\right)+(\frac{EA_{3}}{L_{3}}s^{2}+\frac{12EI_{3}}{L_{3}^{3}}c^{2})\right]+v_{4}^{\Delta T}\left(-\frac{EA_{3}}{L_{3}}s^{2}-\frac{12EI_{3}}{L_{3}^{3}}c^{2}\right)=\alpha \Delta {\mathrm{TEA}}_{2}s\;-\;\alpha \Delta {\mathrm{TEA}}_{3}s \end{array}$$

(A19b)
$$u_{2}^{\Delta T}\left(L\right)+v_{2}^{\Delta T}\left(R\right)+u_{3}^{\Delta T}\left[K+B\right]\;+v_{3}^{\Delta T}\left[M+C\right]+v_{4}^{\Delta T}\left(P\right)=\alpha \Delta {\mathrm{TEA}}_{2}s\;-\;\alpha \Delta {\mathrm{TEA}}_{3}s$$

(A20a)
$$u_{3}^{\Delta T}\left(-\frac{EA_{3}}{L_{3}}cs+\frac{12EI_{3}}{L_{3}^{3}}cs\right)+v_{3}^{\Delta T}\left(-\frac{EA_{3}}{L_{3}}s^{2}-\frac{12EI_{3}}{L_{3}^{3}}c^{2}\right)+v_{4}^{\Delta T}\left(\frac{EA_{3}}{L_{3}}s^{2}+\frac{12EI_{3}}{L_{3}^{3}}c^{2}\right)=\alpha \Delta {\mathrm{TEA}}_{3}s$$

(A20b)
$$u_{3}^{\Delta T}\left(A\right)+v_{3}^{\Delta T}\left(P\right)+v_{4}^{\Delta T}\left(C\right)=\alpha \Delta {\mathrm{TEA}}_{3}s$$

Using Mathematica software, we could achieve the following closed-form expression:

(A21) $$\begin{array}{c} v^{\Delta T}=v_{v}^{4}\\ \left(\frac{1}{C}\right)E\left(\left(\frac{\left(-A_{1}P+A_{2}P+A_{3}R\right)s}{R}+\frac{\left(A_{1}-A_{2}\right)\left(LP-AR\right)\left(cR-Ls\right)}{R\left(-L^{2}+OR\right)}-\frac{\left(AL\left(C+M\right)+\left(F+K\right)LP-\left(C+M\right)OP-A\left(F+K\right)R\right)\left(\left(-L^{2}+OR\right)\left(\left(A_{1}-A_{2}\right)\left(C\left(C+M\right)-P^{2}\right)+\left(-A_{2}C+A_{3}\left(C+P\right)\right)R\right)s-\left(A_{1}-A_{2}\right)\left(C^{2}L+CLM-C\left(F+K\right)R+P\left(-LP+AR\right)\right)\left(cR-Ls\right)\right)}{\left(L^{2}-OR\right)\left(\left(L\left(C+M\right)-\left(F+K\right)R\right)\left(-CL\left(C+M\right)+LP^{2}+C\left(F+K\right)R-APR\right)+\left(-L^{2}+OR\right)\left(\left(C+M\right)\left(-C\left(C+M\right)+P^{2}\right)+CR^{2}\right)\right)}\right)\right)\\ +\left(\left(-AFL-AKL-DLP-LNP+FOP+KOP+ADR+ANR+\frac{\left(AL\left(C+M\right)+\left(F+K\right)LP-\left(C+M\right)OP-A\left(F+K\right)R\right)\left(\left(\left(F+K\right)\left(-C\left(C+M\right)+P^{2}\right)+CLR\right)\left(-L^{2}+OR\right)-\left(-\left(F+K\right)L+\left(D+N\right)R\right)\left(-CL\left(C+M\right)+LP^{2}+C\left(F+K\right)R-APR\right)\right)}{\left(L\left(C+M\right)-\left(F+K\right)R\right)\left(-CL\left(C+M\right)+LP^{2}+C\left(F+K\right)R-APR\right)+\left(-L^{2}+OR\right)\left(\left(C+M\right)\left(-C\left(C+M\right)+P^{2}\right)+CR^{2}\right)}\right)\right)\\ =\frac{\left(\begin{array}{c} \left(-\left(-\left(C+M\right)\left(C\left(F+K\right)-AP\right)+CLR\right)\left(-L^{2}+OR\right)-\left(L\left(C+M\right)-\left(F+K\right)R\right)\left(-C\left(F+K\right)L+C\left(D+N\right)R+A\left(LP-AR\right)\right)\right)\left(\left(-L^{2}+OR\right)\left(\left(A_{1}-A_{2}\right)\left(C\left(C+M\right)-P^{2}\right)+\left(-A_{2}C+A_{3}\left(C+P\right)\right)R\right)s-\left(A_{1}-A_{2}\right)\left(C^{2}L+CLM-C\left(F+K\right)R+P\left(-LP+AR\right)\right)\left(cR-Ls\right)\right)\\ +\left(\left(L\left(C+M\right)-\left(F+K\right)R\right)\left(-CL\left(C+M\right)+LP^{2}+C\left(F+K\right)R-APR\right)+\left(-L^{2}+OR\right)\left(\left(C+M\right)\left(-C\left(C+M\right)+P^{2}\right)+CR^{2}\right)\right)\left(\left(-A_{1}+A_{2}\right)\left(C\left(F+K\right)L-C\left(D+N\right)R+A\left(-LP+AR\right)\right)\left(cR-Ls\right)+\left(-L^{2}+OR\right)\left(\left(-A_{1}+A_{2}\right)\left(-C\left(F+K\right)+AP\right)s+R\left(-A_{2}cC+A_{3}cC+AA_{3}s\right)\right)\right) \end{array}\right)}{(\begin{array}{c} \left(L^{2}-OR\right)\left(\left(\left(F+K\right)\left(-C\left(C+M\right)+P^{2}\right)+CLR\right)\left(-L^{2}+OR\right)-\left(-\left(F+K\right)L+\left(D+N\right)R\right)\left(-CL\left(C+M\right)+LP^{2}+C\left(F+K\right)R-APR\right)\right)\left(\left(\left(C+M\right)\left(C\left(F+K\right)-AP\right)-CLR\right)\left(-L^{2}+OR\right)-\left(L\left(C+M\right)-\left(F+K\right)R\right)\left(-C\left(F+K\right)L+C\left(D+N\right)R+A\left(LP-AR\right)\right)\right)+\\ \left(\left(L\left(C+M\right)-\left(F+K\right)R\right)\left(-CL\left(C+M\right)+LP^{2}+C\left(F+K\right)R-APR\right)+\left(-L^{2}+OR\right)\left(\left(C+M\right)\left(-C\left(C+M\right)+P^{2}\right)+CR^{2}\right)\right)\left(\left(-C{\left(F+K\right)}^{2}+A\left(F+K\right)P+CJR\right)\left(-L^{2}+OR\right)-\left(\left(F+K\right)L-\left(D+N\right)R\right)\left(C\left(F+K\right)L-C\left(D+N\right)R+A\left(-LP+AR\right)\right)\right) \end{array})\alpha \Delta T} \end{array}$$

The response of two inclined beams subject to an external force (F) applied to the central shuttle along the *y*-direction, can be obtained similarly. The equation can be obtained starting from the governing system of Equation (A14), where the thermal load on the right side is substituted with external force (F/2). Then, we obtain:

(A22) $$\begin{array}{l} v^{F_{4}}=v_{v}^{4}\\ =\frac{\left(\frac{F_{e}}{2E}\left(\begin{array}{c} F^{4}+4F^{3}K+6F^{2}K^{2}+4FK^{3}+K^{4}-2F^{2}L^{2}-4FKL^{2}-2K^{2}L^{2}+L^{4}-2DF^{2}M-4DFKM-2DK^{2}M+2FJLM+2JKLM-2DL^{2}M+D^{2}M^{2}-2F^{2}MN-4FKMN-2K^{2}MN-2L^{2}MN+2DM^{2}N+M^{2}N^{2}+2FLMO+2KLMO-JM^{2}O\\ +C^{2}\left({\left(D+N\right)}^{2}-JO\right)+2C\left(JKL+D^{2}M-F^{2}N-2FKN-K^{2}N-L^{2}N+MN^{2}-D\left({\left(F+K\right)}^{2}+L^{2}-2MN\right)+KLO-JMO+FL\left(J+O\right)\right)+DF^{2}P-F^{3}P+F^{2}JP+2DFKP-3F^{2}KP+2FJKP+DK^{2}P-3FK^{2}P+JK^{2}P-K^{3}P-2DFLP+F^{2}LP\\ -FJLP-2DKLP+2FKLP-JKLP+K^{2}LP+DL^{2}P+FL^{2}P+KL^{2}P-L^{3}P-D^{2}MP+DFMP-FJMP+DKMP-JKMP+DLMP+F^{2}NP+2FKNP+K^{2}NP-2FLNP-2KLNP+L^{2}NP-2DMNP+FMNP+KMNP+LMNP-MN^{2}P-FLOP-KLOP\\ +L^{2}OP+JMOP-LMOP+C\left(-D^{2}-JK+D\left(F+K+L-2N\right)+KN+LN-N^{2}+F\left(-J+N\right)+JO-LO\right)P-\left(-4DKL-4KLN+K^{2}O+L^{2}O+F^{2}\left(J+O\right)-\left(D+N\right)\left(D-K-L+N\right)P-KOP+J\left(K^{2}+L^{2}-LP+OP\right)+F\left(2K\left(J+O\right)-OP+\left(D+N\right)\left(-4L+P\right)\right)\right)R\\ -\left({\left(D+N\right)}^{2}-JO\right)R^{2}-A\left(-{\left(F+K-L\right)}^{2}+\left(D-J+N\right)\left(C+M-R\right)\right)\left(C-F-K-L+M+R\right) \end{array}\right)\right)}{\left(\begin{array}{c} C^{3}\left({\left(D+N\right)}^{2}-JO\right)+2C^{2}\left(JKL+D^{2}M-F^{2}N-2FKN-K^{2}N-L^{2}N+MN^{2}-D\left({\left(F+K\right)}^{2}+L^{2}-2MN\right)+KLO-JMO+FL\left(J+O\right)\right)+\left(-JKL-D^{2}M+F^{2}N+2FKN+K^{2}N+L^{2}N-MN^{2}+D\left({\left(F+K\right)}^{2}+L^{2}-2MN\right)-KLO+JMO-FL\left(J+O\right)\right)P^{2}+AP\\ \left(-2F^{3}-6F^{2}K-6FK^{2}-2K^{3}+2KL^{2}+2DKM-JLM+2KMN+2C\left(F+K\right)\left(D+N\right)+2F\left(L^{2}+M\left(D+N\right)\right)-LMO-CL\left(J+O\right)+JKR-2DLR-2LNR+KOR+F\left(J+O\right)R\right)+A^{2}\left(-\left(C+M\right)\left(-{\left(F+K\right)}^{2}-L^{2}+\left(C+M\right)\left(D+N\right)\right)-2\left(F+K\right)LR+\left(D+N\right)R^{2}\right)+C\\ \left(\begin{array}{c} F^{4}+4F^{3}K+K^{4}+L^{4}-2DL^{2}M+D^{2}M^{2}-2L^{2}MN+2DM^{2}N+M^{2}N^{2}-JM^{2}O-D^{2}P^{2}-2DNP^{2}-N^{2}P^{2}+JOP^{2}-L^{2}\left(J+O\right)R-\left({\left(D+N\right)}^{2}-JO\right)R^{2}+2KL\left(M\left(J+O\right)+2\left(D+N\right)R\right)+F^{2}\left(6K^{2}-2\left(L^{2}+M\left(D+N\right)\right)-\left(J+O\right)R\right)-K^{2}\left(2\left(L^{2}+M\left(D+N\right)\right)+\left(J+O\right)R\right)\\ +2F\left(2K^{3}-2K\left(L^{2}+M\left(D+N\right)\right)+LM\left(J+O\right)+2L\left(D+N\right)R-K\left(J+O\right)R\right) \end{array}\right) \end{array}\right)} \end{array}$$

The ratio $K_{A}$ = $F/v^{F}$ represents the stiffness of the v-shaped thermal actuator beam. Therefore, the equation will be:

(A23) $$\begin{array}{l} K_{A}=\\ \frac{2mE\left(\begin{array}{c} C^{3}\left({\left(D+N\right)}^{2}-JO\right)+2C^{2}\left(JKL+D^{2}M-F^{2}N-2FKN-K^{2}N-L^{2}N+MN^{2}-D\left({\left(F+K\right)}^{2}+L^{2}-2MN\right)+KLO-JMO+FL\left(J+O\right)\right)+\left(-JKL-D^{2}M+F^{2}N+2FKN+K^{2}N+L^{2}N-MN^{2}+D\left({\left(F+K\right)}^{2}+L^{2}-2MN\right)-KLO+JMO-FL\left(J+O\right)\right)P^{2}+AP\\ \left(-2F^{3}-6F^{2}K-6FK^{2}-2K^{3}+2KL^{2}+2DKM-JLM+2KMN+2C\left(F+K\right)\left(D+N\right)+2F\left(L^{2}+M\left(D+N\right)\right)-LMO-CL\left(J+O\right)+JKR-2DLR-2LNR+KOR+F\left(J+O\right)R\right)+A^{2}\left(-\left(C+M\right)\left(-{\left(F+K\right)}^{2}-L^{2}+\left(C+M\right)\left(D+N\right)\right)-2\left(F+K\right)LR+\left(D+N\right)R^{2}\right)+C\\ \left(\begin{array}{c} F^{4}+4F^{3}K+K^{4}+L^{4}-2DL^{2}M+D^{2}M^{2}-2L^{2}MN+2DM^{2}N+M^{2}N^{2}-JM^{2}O-D^{2}P^{2}-2DNP^{2}-N^{2}P^{2}+JOP^{2}-L^{2}\left(J+O\right)R-\left({\left(D+N\right)}^{2}-JO\right)R^{2}+2KL\left(M\left(J+O\right)+2\left(D+N\right)R\right)+F^{2}\left(6K^{2}-2\left(L^{2}+M\left(D+N\right)\right)-\left(J+O\right)R\right)-K^{2}\left(2\left(L^{2}+M\left(D+N\right)\right)+\left(J+O\right)R\right)\\ +2F\left(2K^{3}-2K\left(L^{2}+M\left(D+N\right)\right)+LM\left(J+O\right)+2L\left(D+N\right)R-K\left(J+O\right)R\right) \end{array}\right) \end{array}\right)}{\left(\begin{array}{c} F^{4}+4F^{3}K+6F^{2}K^{2}+4FK^{3}+K^{4}-2F^{2}L^{2}-4FKL^{2}-2K^{2}L^{2}+L^{4}-2DF^{2}M-4DFKM-2DK^{2}M+2FJLM+2JKLM-2DL^{2}M+D^{2}M^{2}-2F^{2}MN-4FKMN-2K^{2}MN-2L^{2}MN+2DM^{2}N+M^{2}N^{2}+2FLMO+2KLMO-JM^{2}O\\ +C^{2}\left({\left(D+N\right)}^{2}-JO\right)+2C\left(JKL+D^{2}M-F^{2}N-2FKN-K^{2}N-L^{2}N+MN^{2}-D\left({\left(F+K\right)}^{2}+L^{2}-2MN\right)+KLO-JMO+FL\left(J+O\right)\right)+DF^{2}P-F^{3}P+F^{2}JP+2DFKP-3F^{2}KP+2FJKP+DK^{2}P-3FK^{2}P+JK^{2}P-K^{3}P-2DFLP+F^{2}LP\\ -FJLP-2DKLP+2FKLP-JKLP+K^{2}LP+DL^{2}P+FL^{2}P+KL^{2}P-L^{3}P-D^{2}MP+DFMP-FJMP+DKMP-JKMP+DLMP+F^{2}NP+2FKNP+K^{2}NP-2FLNP-2KLNP+L^{2}NP-2DMNP+FMNP+KMNP+LMNP-MN^{2}P-FLOP-KLOP\\ +L^{2}OP+JMOP-LMOP+C\left(-D^{2}-JK+D\left(F+K+L-2N\right)+KN+LN-N^{2}+F\left(-J+N\right)+JO-LO\right)P-\left(-4DKL-4KLN+K^{2}O+L^{2}O+F^{2}\left(J+O\right)-\left(D+N\right)\left(D-K-L+N\right)P-KOP+J\left(K^{2}+L^{2}-LP+OP\right)+F\left(2K\left(J+O\right)-OP+\left(D+N\right)\left(-4L+P\right)\right)\right)R\\ -\left({\left(D+N\right)}^{2}-JO\right)R^{2}-A\left(-{\left(F+K-L\right)}^{2}+\left(D-J+N\right)\left(C+M-R\right)\right)\left(C-F-K-L+M+R\right) \end{array}\right)} \end{array}$$

where m is the number of thermal actuator beams.
